# Evolution of the Sex Pheromone Communication System in *Ostrinia* Moths

**DOI:** 10.3390/insects12121067

**Published:** 2021-11-28

**Authors:** Dan-Dan Zhang

**Affiliations:** Department of Biology, Lund University, 22362 Lund, Sweden; dan-dan.zhang@biol.lu.se

**Keywords:** biosynthetic pathway, pheromone diversification, pheromone receptor (PR) genes, olfactory sensory neurons (OSNs), antennal lobe, asymmetric tracking, behavioural antagonists, broadly tuned receptors

## Abstract

**Simple Summary:**

Moths typically rely on sex pheromone communication to find a mate. This involves the production of species-specific sex pheromones by females (the signaller) and the corresponding selective detection by conspecific males (the receiver). A key question in the evolution of the pheromone communication system is how the female signals can diversify and still be tracked by the receivers over the process of speciation. The genus *Ostrinia*, which comprises 20 species worldwide including several well-recognised agricultural pests, is an attractive model in the study of the evolution of sex pheromone communication, as the closely related species and strains provide an ideal example of ongoing speciation. This review presents a comprehensive overview of the research on pheromone communication in different *Ostrinia* species over the past four decades, from the identity and biosynthesis of pheromones in the females to the molecular and neuronal basis of the pheromone perception in males. The evolutionary insights from these studies are discussed and the directions for future research are outlined.

**Abstract:**

It remains a conundrum in the evolution of sexual communication how the signals and responses can co-ordinate the changes during speciation. The genus *Ostrinia* contains several closely related species as well as distinctive strains with pheromone polymorphism and represents an example of ongoing speciation. Extensive studies in the genus, especially in the species the European corn borer *O. nubilalis* (ECB), the Asian corn borer *O. furnacalis* (ACB) and the adzuki bean borer *O. scapulalis* (ABB), have provided valuable insights into the evolution of sex pheromone communication. This review presents a comprehensive overview of the research on pheromone communication in different *Ostrinia* species over the past four decades, including pheromone identification and biosynthesis, the ligand profiles of pheromone receptor (PR) genes, the physiology of peripheral olfactory sensory neurons (OSNs) and the projection pattern to the antennal lobe. By integrating and comparing the closely related *Ostrinia* species and strains, it provides an evolutionary perspective on the sex pheromone communication in moths in general and also outlines the outstanding questions that await to be elucidated by future studies.

## 1. Introduction

It remains enigmatic in the evolution of sexual communication how signals diversify under strong stabilizing selection in general and how the changes in signals and responses can co-ordinate despite the fact that the genes controlling the production and the response of the signals are normally unlinked [1,2]. The moth sex pheromone communication system provides an unprecedented opportunity to elucidate this question, as major advances have been made to understand the diversity of the signal production in females, down to the changes in the underlying biosynthetic pathway, as well as the molecular and neural basis of the signal reception in males.

The diversity of the female-produced sex pheromone can evolve through amino acid mutations in the coding region of related biosynthetic genes or through regulation of gene expressions; it can involve either a small number of genes or many genes (polygenic changes) [3,4,5,6,7,8]. More recently, accumulating data from functional analysis of the pheromone receptor (PR) genes has allowed us to infer on the molecular level how the males track changes in female sex pheromones and how the evolution on signallers’ and receivers’ sides co-ordinate [9].

Due to the potential value in pest control, there have been extensive studies on *Ostrinia* moths, which revealed intriguing features of pheromone diversification and perception and thus makes the *Ostrinia* species an attractive model in the field of chemical ecology for the study of the evolution of sex pheromone communication. This paper presents a comprehensive overview of the current status of the research on pheromone communication in different *Ostrinia* species. Particular emphasis is put on the expression and ligand affinities of PR genes in the peripheral olfactory sensory neurons (OSNs) and the projection pattern to the antennal lobe. By integrating and comparing the systems in closely related *Ostrinia* species and strains, it will provide an evolutionary perspective on the sex pheromone communication in moths.

## 2. The *Ostrinia* Species and Female Sex Pheromones

**Figure 1:**
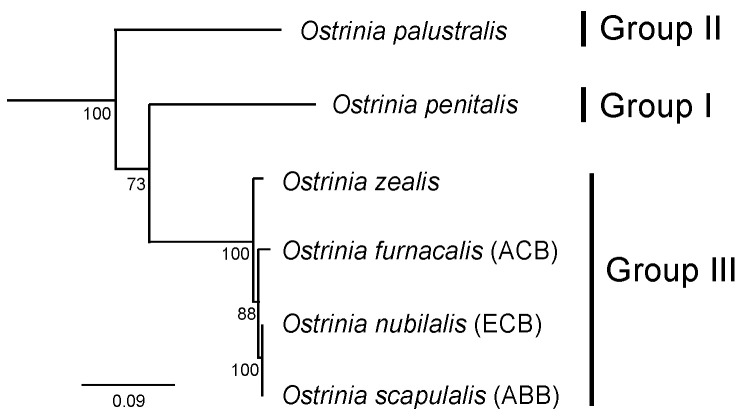
The phylogeny of six *Ostrinia* species, adapted from [14]. The maximum likelihood tree was built based on 13 protein-coding genes from the mitogenomes. Percentage bootstrap support (N = 1000) values are shown at corresponding nodes. Scale bar indicates nucleotide substitutions per site. The classification of groups according to the number of uncus lobes in male genitalia are labelled on the right side.

The genus *Ostrinia* comprises 20 species worldwide [10], several of which are well-recognised agricultural pests, such as the pests of maize, *O. nubilalis* and *O. furnacalis*. These species can be further classified into three groups according to the number of uncus lobes in male genitalia, i.e., simple uncus group (Group I), bilobed uncus group (Group II) and trilobed uncus group (Group III) [10,11,12] (Figure 1). The trilobed uncus group includes ten species that are confusingly similar in appearance but vary in the morphology of their male mid-tibia, i.e., small, medium or massive [13]. Nevertheless, the male mid-tibia morphology is not a reliable character for species definition, whereas reproductive isolation is strongly related to host-plant type. Consequently, it was proposed that *O. narynensis* and *O. orientalis* are synonymized with *O. scapulalis*; *O. nubilalis* feeding on mugwort, hop and several other dicotyledons also belongs to *O. scapulalis* while only *O. nubilalis* feeding on maize belongs to *O. nubilalis* [13].

The sex pheromones of nine *Ostrinia* species have been identified (Pherobase). Among these, the European corn borer *O. nubilalis* (ECB), the Asian corn borer *O. furnacalis* (ACB) and the adzuki bean borer *O. scapulalis* (ABB) which all belong to the trilobed uncus Group III [10] are most intensively investigated (Figure 1). ECB distributes across North America, Europe and North Africa, while ACB occurs in the East Palearctic, the Oriental and part of the Australian regions, suggesting them as two allopatric species. ABB occurs only in Palearctic region and the populations overlap with those of ECB and ACB [13]. Strong but incomplete reproductive isolation exists between ABB and ECB, representing a case of ongoing speciation [15,16].

**Figure 2:**
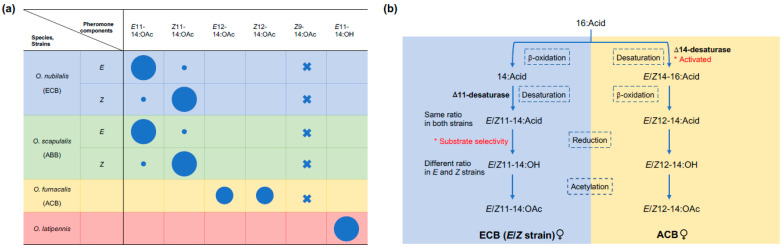
The pheromone composition (**a**) and biosynthetic pathway (**b**) in *Ostrinia* moths. The size of circles in (**a**) roughly correspond to the blend ratio, and the crossings represent behavioural antagonist. The key steps in the pathway that cause the shift in the pheromone composition are highlighted with asterisks in (**b**).

ECB and ABB utilise (*E*)-11-tetradecenyl acetate (*E*11) and (*Z*)-11-tetradecenyl acetate (*Z*11) as sex pheromone components, like most of the other *Ostrinia* species. In addition, both species exhibit intraspecific polymorphism in the relative proportion of the components; *E*11 is the major component in the *E*-strain while *Z*11 is dominant in the *Z*-strain [17,18,19] (Figure 2a). The *E* and *Z* strains, sometimes also referred to as races, are indistinguishable based on the morphology or the common barcoding genes such as cytochrome c oxidase I (COI) and cytochrome B (CytB); they are however distinguishable by the pheromone biosynthesis genes (fatty-acyl reductase and Δ11-desaturase genes) [20]. They may have the same or different host plant preference [21,22,23]. For ECB, the *Z* strain is the most prevalent pheromone phenotype, while the *E* strain has a more restricted distribution [24,25]; the two strains occur sympatrically at several locations within their range. Hybridization could occur under laboratory condition or in limited regions where the two strains are in sympatry, and does not cause obvious reduction of fitness. In contrast, the *E* and *Z* strains of ACB are sympatric in most regions of Japan, and the population with intermediate pheromone blend proportions are dominant, indicating a higher frequency of hybridization [26]. The genetic divergence between the two strains is weak yet suggestive of ongoing speciation [17,27].

ACB is thought to have diverged from the common ancestor with ECB around one million years ago and is unique within *Ostrinia*, using the novel pheromone blend (*E*)-12-tetradecenyl acetate (*E*12) and (*Z*)-12-tetradecenyl acetate (*Z*12) [28] (Figure 2a). (*Z*)-9-tetradecenyl acetate (*Z*9), which is a constituent of the pheromones used by the two species *O. zealis* and *O. zaguliaevi* [29,30], is the common behavioural antagonist of the above-mentioned three species [30,31,32]. The more ancestral bifid uncus Group II includes the Far Eastern knotweed borer *O. latipennis*, which uses (*E*)-11-tetradecenol (*E*11-14:OH) as its single pheromone component [33] (Figure 2a).

## 3. The Molecular Basis of Pheromone Diversification

The pheromone components in ECB and ACB are produced through the biosynthetic pathway (as shown in Figure 2b) which is derived from normal fatty acid metabolism with changes in tissue specificity and function [4]. Mutation or regulation of the genes encoding relevant biosynthetic enzymes plays a key role in the diversity of pheromone signals.

The desaturases constitute a multigene family which undergoes birth-and-death evolution with multiple gene duplication and nonfunctionalization events. The shift from *E*11- and *Z*11- to *E*12- and *Z*12- in ACB takes place in the desaturation step, when Δ14-desaturase gene is activated and produces Δ14-16:Acids. Through one step of limited β-oxidation, Δ14-16:Acids are then chain shortened to *E*/*Z*12-14:Acids which serve as precursors of ACB sex pheromones (Figure 2b) [3,4]. The transcripts of three different desaturase genes (Δ9-, Δ11- and Δ14-) are present in both ACB and ECB. However, in female ACB only Δ14-desaturase gene is active whereas Δ11- desaturase gene is non-functional; conversely, in female ECB only Δ11-desaturase gene is active whereas Δ14-desaturase gene is non-functional [3,4]. Therefore, the saltational change involving the activation of Δ14-desaturase gene in ACB which was nonfunctional in the ancestral *Ostrinia* species causes the major shift in the pheromone [3,4]. It is yet unknown whether the activation and non-functionalization of related desaturase genes are underlain by epigenetic mechanism such as translational repression or by a few amino acid substitutions in the coding region.

**Figure 3:**
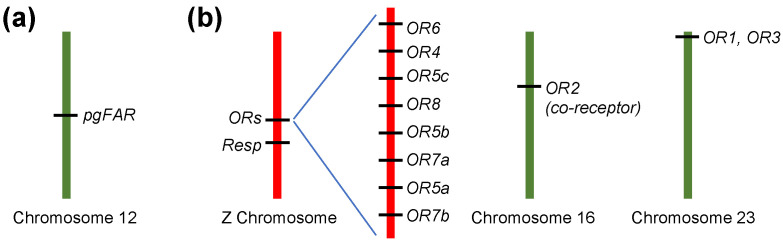
The production of female sex pheromones and male reception are physically unlinked in the European corn borer *O. nubilalis* (ECB), controlled by genes on different chromosomes. (**a**) The reversed ratio of pheromones in *E* and *Z* strains is controlled by one autosomal locus on chromosome 12 [35,36,37], which encodes fatty-acyl reductase in pheromone gland (*pgFAR*) with strain specific substrate specificities [7]. (**b**) The male response is controlled by a sex-linked quantitative trait locus (QTL), *Resp,* and an autosomal locus not linked to the female production locus [6]. The autosomal locus might correspond to the array of OR1 and OR3 located on chromosome 23 [37,39], whereas the sex-linked *Resp* locus is 15 cm distant from the large cluster comprised of eight PR genes at Z chromosome [40,41]. The co-receptor Orco (previously referred to as OR2) gene is located on chromosome 16 [39]. The total number of chromosomes in *Ostrinia* spp. is 31 [42].

The reversed ratio of *E*11- and *Z*11- in *E* and *Z* strains of ECB is due to different substrate selectivities of their respective reductases [34], as a result of the allelic variation in the encoding pheromone gland specific fatty-acyl reductase gene (*pgFAR*) in the two strains [7]. This is consistent with previous findings that the difference in the pheromone production of ECB *E* and *Z* strains is controlled by one autosomal locus with two alleles (Figure 3) [35,36,37]. Nevertheless, it should be noted that there is a significant variation in the *E*/*Z* ratio among both strains under nature conditions which could not be directly explained by the basic one-locus two-allele model. There might be polymorphism at the locus in natural populations [38]. It was proposed that the basic model can be extended as there being two different alleles in the *Z* strain along with the *E* strain allele at the major pheromone production locus, and that there is an additional locus independent from the major locus [38].

## 4. Male Produced Pheromones

In addition to the female sex pheromones, the male produced pheromones associated with hairpencil display were also identified from the *Ostrinia* moths which seemed to be species specific. The pheromone of male ECB *Z* strain was characterized as a blend of 16 carbon acetates, including hexadecanyl acetate (16:OAc) and (*Z*)-9-, (*Z*)-11- and (*Z*)-14-hexadecenyl acetates (*Z*9-16:OAc, *Z*11-16:OAc and *Z*14-16:OAc), whereas *Z*11-16:OAc is of low abundance or completely absent in the *E* strain; male ACB only produces 16:OAc and *Z*9-16:OAc, with *Z*14-16:OAc missing compared to ECB [43]. The male pheromones may reinforce the reproductive isolation among different *Ostrinia* species/strains. Furthermore, the composition of the male pheromones in ECB *Z* strain was related to age, which might be an indicator of the quality of male moths and is likely to be correlated with the females’ mating preference to older males [43].

It was proposed that the male *Ostrinia* pheromones are produced by a biosynthetic pathway similar to that in females, which involves Δ9-, Δ11- and Δ14-desaturation [43]. The Δ14-desaturase gene that is nonfunctional in female ECB might be always active in male ECB taking part in male pheromone synthesis and has changed to be expressed in females in ACB. Thus, the genetic correlation between male and female pheromones might facilitate the rapid evolution of new signals by saltational changes [43].

## 5. Ligand Profiles of Pheromone Receptor Genes in Males

Male moths detect the female-released pheromones by the PR genes expressed in dendrite of OSNs housed in sensilla trichodea on their antennae [44]. Almost all the *Ostrinia* PR genes are exclusively expressed in male antennae, although OR1 and OR7 are also expressed at very low levels in female antennae [45,46,47,48]. The first PR gene deorphanized from *Ostrinia* species was OR1 that was conserved across the genus [49]. OR1 in *O. latipennis* was responsive to its pheromone component *E*11-14:OH and the orthologue in ABB also responded specifically to *E*11-14:OH although the species does not use this ancestral pheromone compound. ABB males also showed antennal responses to *E*11-14:OH, thus the compound might have an unknown function, such as antagonist, in the sex pheromone communication of ABB that has not been tested to dated [49].

**Figure 4:**
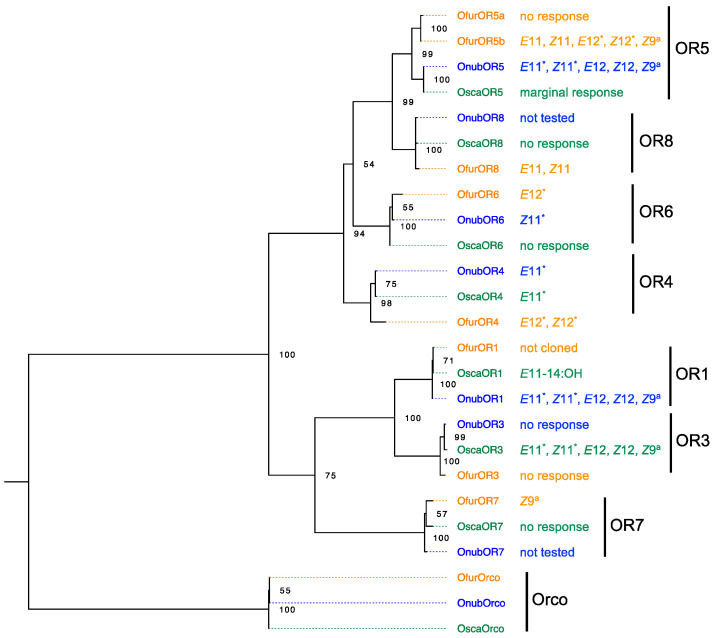
The phylogeny of PR genes in *Ostrinia* species and their ligand profiles. The amino acid sequences (Table S1) were aligned with MUSCLE [51]. The maximum likelihood tree rooted with the Orco lineage was constructed using MEGA X [52,53] with LG + G substitution model [54]. Percentage bootstrap support (500 replicates) values over 50 are labelled at corresponding nodes. Bule, orange and green colours represent ECB, ACB and ABB respectively. * indicates the major pheromone components in respective species; ^a^ indicates the behavioural antagonist. Nomenclature of the OnubORs follows the references [39,45,47,49,55]. In this name system, OR1, OR3, OR4 and OR5 in ECB correspond to OnubOR5, OnubOR4, OnubOR3 and OnubOR1 referred in [46,50].

Since then, more orthologous clusters of PRs have been identified in the genus, i.e., OR3-OR8 (Figure 4). Among these receptor genes, OR4 had a relatively specific response to the pheromone component *E*11 in ECB and ABB. OR6 responded specifically to the other pheromone component *Z*11 in ECB whereas no response was found to the tested compounds in ABB. OnubOR1, OnubOR5a and OscaOR3 had broad response spectra, responding not only to their own pheromone components *E*11 and *Z*11 but also to the pheromone components for ACB *E*12 and *Z*12 as well as the behavioural antagonist *Z*9. The other receptors had no response or marginal responses to the tested compounds [45,46,50].

Interestingly, the PR genes of ECB are in tandem arrays, possibly formed by gene duplication during the birth-and-death evolution; the large cluster comprised of eight PR genes (OR7b-OR5a-OR7a-OR5b-OR8-OR5c-OR4-OR6) is at a locus on Z chromosome; the array of OR1 and OR3 is located on chromosome 23 (Figure 3) [37,39]. This is in agreement with previous findings that the male response is controlled by a sex-linked quantitative trait locus (QTL) and an autosomal locus not linked to the female production locus [6]. The odorant receptor co-receptor (Orco, previously referred to as OR2) gene, which forms heteromeric complex with conventional ORs and essential for their proper functioning, is located on chromosome 16 (Figure 3) [39].

In ACB, OfurOR4 had a large response to both *E*12 and *Z*12 [50,55] whereas OfurOR6 responded specifically to *E*12 [55]. Therefore, ACB did not evolve a novel set of receptor genes distinct from those for *E*11 and *Z*11 but recruited existing orthologous receptor genes to detect the unique pheromone components *E*12 and *Z*12; the responses to *E*12 and *Z*12 had evolved in parallel in different orthologous clusters. Meanwhile, OfurOR8 was tuned to *E*11 and *Z*11, OfurOR7 responded specifically to the behavioural antagonist *Z*9, OfurOR5b was a broadly tuned receptor, while OfurOR3 and OfurOR5a were ‘silent’ to all the tested compounds [55] (Figure 4).

## 6. Responses of Peripheral Olfactory Sensory Neurons to Pheromone Components

Three subtypes of sensilla trichodea were identified from ECB by morphological investigation and in vivo electrophysiological recordings from single sensilla [56,57,58]. Trichodea type A is longer and innervated by three sensory neurons; in both *E* and *Z* strains, the large-spike neurons respond specifically to corresponding major pheromone component, the small-spike neurons are specific to the minor component and the third neuron to the behavioural antagonist *Z*9. Trichodea type B and C are shorter and house two and one sensory neurons respectively. The neurons in type B respond to pheromone components only, while the single neuron in type C responds to either the major pheromone component or the behavioural antagonist. It was demonstrated that there is a correlation between the spike amplitudes and the dendrite diameter of the neurons, i.e., the OSNs with larger dendrite diameter produce spikes with larger amplitudes [58].

More recently, Koutroumpa et al. [47] reported that there was only one type of sensilla (corresponding to the previously identified trichodea type A) involved in pheromone detection in both strains of ECB. This discrepancy might be due to technical issues; previous studies used the cut-tip technique that might have affected the response sensitivity of the neurons, and higher concentrations that might have caused cross-sensitivity of the receptors and obscured differences in spike amplitude. Koutroumpa et al. also found that the medium spike neurons to behavioural antagonists were broadly tuned, evoked by ten of thirteen compounds including *Z*9 and (*Z*)-11-hexadecenal (*Z*11-16:Ald). It was inferred that OnubOR7 was specific for *Z*11-16:Ald based on its expression pattern restricted to lateral sensilla which coincided with the electrophysiological responses to *Z*11-16:Ald [47].

ABB was thought to have a similar layout of peripheral OSNs as ECB [45], while it seems to be more complicated in ACB, with four physiological types of trichoid sensilla (type 1–4) that were further characterized as four subtypes (subtype A–D) [32]. 97% of the trichoid sensilla (type 1–3) were innervated by three sensory neurons as in ECB and the large-spike neurons responded to both pheromone components *E*12 and *Z*12 in an identical dose-dependent fashion; most of them also house a small-spike neuron that either exclusively responded to or had higher affinity to *E*12. Most of the medium-spike neurons were tuned broadly to the behavioural antagonists or specifically to *Z*9. It was considered to be unusual that the majority of the peripheral OSNs in ACB were devoted to the detection of both pheromone components, although a small proportion of the OSNs responded specifically to either component. This may result in higher sensitivity but lower specificity. As ACB is the only known Lepidopteran species that use the unique pheromone compounds *E*12 and *Z*12, perhaps the demand for specificity is less restrictive [32].

## 7. Antennal Lobe Projection Pattern of Pheromone Specific OSNs

Central nervous processing is the link between peripheral receptor responses and behavioural output, and the antennal lobe (AL) is the first order olfactory processing centre in the insect brain. The glomeruli densely packed in AL receive the olfactory inputs from the peripheral OSNs via antennal nerves (AN) which are in turn relayed to the projection neurons (PN) and projected to the mushroom body and the lateral horn. The other group of neurons in AL, local interneurons (LN), arborize in all or part of the glomeruli. The synaptic interaction among OSNs, PNs and LNs reformats the olfactory information input into a spatio-temporal code that is sent to higher brain centres [59,60].

**Figure 5:**
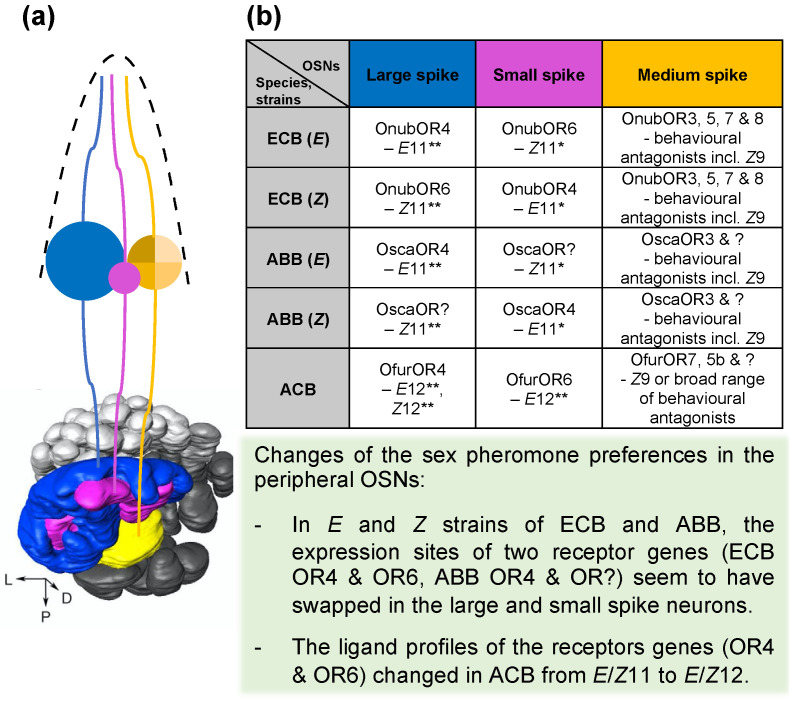
Schematic showing (**a**) the arrangement of peripheral OSNs in the most prevalent type of sensilla trichodea on antennae of male *Ostrinia* moths and the projection to MGC in the antennal lobe, as well as (**b**) the putative location and ligand profiles of pheromone receptor genes in the OSNs. ** and * indicate the major and minor pheromone components in respective species/strains. The sensillum depicted here corresponds to sensilla trichodea type A in both strains of ECB and ABB, and sensilla trichodea type 1 and type 2 (subtype A–C) in ACB. The layout of the circuit is identical in all these three *Ostrinia* species including *E* and *Z* strains, with the large spike neurons responsive to the primary pheromone component projecting to the medial glomerulus, the small spike neurons responsive to the secondary pheromone component projecting to the lateral glomerulus and the medium spike neurons responsive to the behavioural antagonists to the posterior glomerulus. Illustration in (**a**) is adapted from [47,62].

Males of both strains of ECB have ~66 glomeruli in AL, with morphologically indistinguishable macroglomerular complex (MGC) at the entrance of AN consisting of three enlarged glomeruli; a larger medial glomerulus and a smaller lateral glomerulus that are interdigitated, and a posterior disc-shaped one. In both strains, the large spike OSNs project to the medial glomerulus, the small spike OSNs to the lateral glomerulus, while the medium spike neurons to the posterior glomerulus. However, since in *E* strains the large spike OSNs responded to *E*11 and small spike OSNs responded to *Z*11, whereas in *Z* strains the large spike OSNs responded to *Z*11 and small spike OSNs responded to *E*11, the functional topology in the MGC of the two strains was reversed although the wiring remained unchanged (Figure 5a). It is therefore suggested that the shift of pheromone preference occurred upstream of AL, i.e., in the peripheral circuit where the neuronal identities of the large spike and small spike OSNs interchanged [61,62].

## 8. The Location of Pheromone Receptor Genes in OSNs

By linking the in vitro response profiles of PR genes and the sensory physiology of OSNs, we could putatively assign the PR genes of the ECB *E* and *Z* strains to particular OSNs in the sensilla (Figure 5b). In the *E* strain, the large spike neurons in sensilla trichodea type A responsive to *E*11 express OR4 and the small spike neurons responsive to *Z*11 express OR6. Interestingly, in the *Z* strain OR4 and OR6 seem to have swapped their expression sites, thus the large spike neurons respond to *Z*11 and the small spike neurons respond to *E*11 [47,50]. The swapping of receptor gene expression sites could account for the interchange of neuronal identities proposed by Karpati et al. [62]. Furthermore, both strains have the medium spike neurons that broadly respond to the behavioural antagonists including *Z*9 and co-express up to four PR genes in addition to the co-receptor Orco, which represents a prominent exception to the canonical one receptor-one neuron rule [47].

It’s likely that the expression pattern of PR genes in OSNs of ABB and ECB is similar (Figure 5) since they have identical pheromone communication systems [45]. However, the PR gene specific to *Z*11 has not been found in ABB, it is therefore not clear which gene corresponds to the small spike neurons of *E* strain ABB and the large spike neurons of *Z* strain ABB.

In sensilla trichodea type 1 and type 2 of ACB which together represent almost 70% of sensilla trichodea on male antennae, OR4 might correspond to the large spike neurons tuned to *E*12 and *Z*12 while OR6 might correspond to the small spike neurons tuned to *E*12 specifically. The broadly tuned OR5b and the specific *Z*9 receptor OR7 are likely to be co-expressed in the medium spike neurons of subtype A and subtype B sensilla, while the medium spike neurons in subtype C sensilla might express OR7 alone (Figure 5b). Compared to *E* strain of ECB, OR4 and OR6 in ACB do not change their expression sites, but their ligand profiles of the genes altered; OR4 changed from detecting *E*11 specifically to detecting both *E*12 and *Z*12, and OR6 changed from detecting *Z*11 to *E*12. The altered ligand profiles of OR4 and hence the shift of large spiking neuron phenotype in ACB could have resulted from a single mutation in the receptor gene [50].

It should be noted that the proposed model in Figure 5 is a simplified schematic of the most prevalent sensilla type and corresponding innervation pattern in respective species, and the condition is much more complicated in *Ostrinia* moths, especially in ACB where many different sensilla types are present on the antennae.

## 9. The Evolutionary Perspective

The pheromone communication system of moths is generally under stabilizing selection to ensure the efficiency of mate selection. The conundrum is how signals can diverge which lead to speciation. This is particularly challenging as genes controlling female pheromone production and male behavioral response are genetically unlinked (Figure 3). Extensive studies over the past four decades may help us to resolve the evolutionary trajectories of the *Ostrinia* species.

### 9.1. On the Signalers’ Side

The divergence in the production of female sex pheromones occurred owing to the alteration in related biosynthetic genes. The major shift from *E*11 and *Z*11 to *E*12 and *Z*12 in ACB is by activation of the nonfunctional Δ14 desaturase gene instead of small adaptive changes. The desaturase gene family was supposed to evolve under a birth-and-death model with multiple events of gene duplication, gene loss and pseudolization [3,4]. The variation of sex pheromone composition in *E* and *Z* strains of ECB is due to the substitutions in the coding region of reductase. The specificity of the enzyme is hence modified, contributing to the incomplete reproductive isolation which might lead to eventual speciation [7].

Genes encoding other enzymes crucial for the sex pheromone biosynthesis, such as chain-shortening enzymes and acetyltransferases, of a vast majority of moths have not been functional characterized yet, except for a recent report of two acyl-CoA oxidase genes involved in chain-shortening in the European grapevine moth, *Lobesia botrana* [63]. Further studies are needed to identify genes encoding chain-shortening enzymes and acetyltransferases in *Ostrinia* moths and verify whether any differences in these genes contribute to the variation of pheromone signals.

### 9.2. On the Receivers’ Side

The multigene family of male PRs are generally under strong selection pressure [9,50,64], yet are still be able to track the changes in female pheromone production. The branches leading to ACB in OR4 and OR6 lineages have been relaxed from evolutionary constraints and show evidence of positive selection (with normalized nonsynonymous to synonymous substitution rates greater than 1), which might account for the shift of ligand specificities of the orthologous genes from *E*/*Z*11 to *E*/*Z*12 in ACB [50]. Indeed, a single mutation of a residue in ECB OR4 to the corresponding residue in ACB could significantly reduce its response to *E*11 thus the broader response specificity was narrowed to correspond to *E*12 and *Z*12 [50]. In addition, the OR6 orthologues also exhibit distinct ligand profiles although the amino acid sequence identities are higher than 94%; OR6 respond to *Z*11 in ECB (*Z* strain), to *E*12 in ACB and has no response in ABB. To track the different sex pheromone compositions, the males in *E* and *Z* strains of ECB seem to have swapped the expression sites of OR4 and OR6 in large and small spike neurons [47,50]. Therefore, it might seem that the shifts of pheromones preference in males occur at peripheral level, changing the ligand profiles of the receptor genes or switching the expression sites, and do not involve the rewiring of the existing olfactory circuit [62]. Indeed, the insect antennal lobe is hard-wired and the ORs themselves have no influence over map formation [65,66]. The great flexibility of the peripheral olfactory system enables the fast evolution of pheromone recognition in response to the changing signals.

However, it remains puzzling how the changes on PR level eventually give rise to altered male responses, as the PR cluster on Z chromosome in ECB is around 15 cm distant from *Resp*, the locus responsible for male response. This is different from the case in *Heliothis* moths where a tightly linked cluster of PRs is located in an autosomal QTL directly responsible for male preference [67]. It is possible that a *trans*-acting transcription factor residing in *Resp* region regulates the expression of OR4 and OR6. Another possibility is that the different male preferences are controlled at the central level by changes in axonal targeting. Genes putatively involved in neurogenesis were identified in *Resp* region in *E* and *Z* strains of ECB [40]. Among these, the gene encoding transcription factor *bric à brac* (*bab*) was shown to control male preference by variation in its first intron [41]. *Bab* was supposed to play a role in the development of ECB olfactory system [41]; it remains to be resolved whether *bab* and other genes in *Resp* region function by *trans*-acting effects on the expression of PR genes or by altering the axonal targeting of OSNs to antennal lobe. It is worth noting that variations in *bab* intron 1 and *pg*FAR have strong genomic associations although they are located on different chromosomes [41]. This might explain how divergences in female pheromone signals and male responses coevolve albeit lack of physical linkage of relevant genes.

### 9.3. Broad Response Spectrum of Male Moths

An intriguing characteristic of pheromone reception in male *Ostrinia* moths is the broad response spectrum. Many of their PR genes are broadly tuned and the co-expression of multiple PR genes in the medium spike neurons further broadens the response spectrum [47]. This might be an adaptive advantage for male moths, as the male response should be broad enough to track the variation in female-released pheromones following the asymmetric tracking hypothesis [68,69]. Some rare males in ECB population are less specific in the behavioural response to related pheromone blends and can be attracted to females of a different strain or closely related species (ACB) [70]. This capacity might be imparted by the functioning of broadly tuned receptors.

On the other hand, the *Ostrinia* moths are also under pressure to evolve broad behavioural antagonism to avoid heterospecific mating, as many moth species use *E*11 and *Z*11 as common pheromone components. The co-expression of multiple PR genes in the antagonist responsive neurons may ensure the broad behavioural antagonism. It was considered that a general antagonist detector, i.e., an unspecific neuron responsive to all antagonists, is sufficient to abort the flight of male moths toward the pheromone blend source [32].

### 9.4. Other Hypotheses and Forms of Selection

Above we have discussed the molecular basis of the diversification of pheromone signals. Other than that, there have been some other hypotheses or models on how signals can diversify under stabilizing selection. For example, the “proximity model” proposed that female moths with variable attractiveness to males benefit from calling in close proximity with each other and can thus sustain signal variation [71]; the “experience hypothesis” proposed that female moths may alter their sex pheromone blend according to the prevailing olfactory cues they are exposed to, and such adaptive phenotypic plasticity eventually leads to signal variation [72].

Although moth sex pheromone communication system is under stabilizing selection in general to ensure mate recognition and avoid hybridization, directional selection may occur when there is communication interference from closely related sympatric species. This may counteract intraspecific stabilizing selection and lead to diversification of the signals [73]. For example, *H. virescens* males exert intense directional selection on *H. subflexa* females to produce relatively high amount of acetate esters [74].

Further studies are needed to assess whether other models or forms of selection are involved in the evolution of pheromone communication channels of *Ostrinia* moths.

## 10. Concluding Remark

*Ostrinia* moths have been an ideal model to explore the evolution of sex pheromone communication. The genus includes several closely related species as well as distinctive strains as a result of pheromone polymorphism and represents an example of ongoing speciation. The extensive studies on the pheromone communication system in this genus have provided us with valuable insights. Nevertheless, outstanding questions remain to be addressed by future research.

Biosynthetic genes encoding chain-shortening enzymes and acetyltransferases are to be identified and their contributions to pheromone signal variation need to be evaluated. The site where male pheromones are synthesized remains elusive and the proposed biosynthetic pathway awaits further experimental verification. The responses of female antennae to male pheromones need to be tested; the corresponding sensilla and the receptor genes expressed therein are to be identified.

On the receivers’ side, what remains to be determined is whether the changes in the male preference of ECB *E* and *Z* strains occur at peripheral level or central nervous level. Although the interchange of OR4 and OR6 expression sites seem to be an attractive hypothesis, the question is the PR tandem in the Z chromosome is distant from *Resp* locus. Whether the expression of OR4 and OR6 are *trans*-regulated by *bab* or other transcription factor in *Resp* region remains to be tested. Alternatively, empirical evidence is needed to support the idea that the genes in *Resp* region alter the axonal targeting of OSNs.

The mechanism underlying the reproductive isolation between ECB and ABB is still elusive. It was indicated by QTL mapping that the prezygotic isolation was independent from female pheromone production and male responses [16]. Further studies are needed to determine whether it is related to male pheromone production and the detection by females, or any other factors.

Finally, the evolution of sex pheromone communication channels in *Ostrinia* moths is distinct from, for example, *Heliothis* moths in which the switching of PRs directly causes the changes in the male preference [67]. One should therefore acknowledge the complexity and diversity of moth sex pheromone communication system, as different species may have different evolutionary trajectories.

## Supplementary Materials

The following are available online at https://www.mdpi.com/article/10.3390/insects12121067/s1, Table S1: Genbank accession numbers of the amino acid sequences used in the construction of phylogenetic tree in Figure 4.
