# Supplementary material for: Evolution of the Sex Pheromone Communication System in Ostrinia Moths
Source: Insects. 2021 Nov 28;12(12):1067. doi: 10.3390/insects12121067 (PMC8708824; doi:10.3390/insects12121067)
Supplement: Supplementary file 1 [file insects-12-01067-s001.zip › insects-1453644-supplementary.pdf]

**Table S1.** Genbank accession numbers of the amino acid sequences used in the construction of phylogenetic tree in Figure 4.

| Gene name | Accession no. | Gene name | Accession no. | Gene name | Accession no. |
|---|---|---|---|---|---|
| **ACB** | | **ECB** | | **ABB** | |
| OfurOR1 | AGG91642 | OnubOR1 | ADB89182 | OscaOR1 | BAH57975 |
| OfurOR3 | AGG91644 | OnubOR3 | ADB89181 | OscaOR3 | BAI66604 |
| OfurOR4 | AGG91645 | OnubOR4 | ADB89180 | OscaOR4 | BAI66605 |
| OfurOR5a | AGG91646 | OnubOR5 | ADB89178 | OscaOR5 | BAI66607 |
| OfurOR5b | AGG91647 | OnubOR6 | ADB89183 | OscaOR6 | BAJ22889 |
| OfurOR6 | AGG91648 | OnubOR7 | BAI66627 | OscaOR7 | BAI66609 |
| OfurOR7 | AGG91649 | OnubOR8 | BAJ61934 | OscaOR8 | BAI66610 |
| OfurOR8 | AGG91650 | OnubOrco | ADB89179 | OscaOrco | BAH57973 |
| OfurOrco | AGG91643 | | | | |
